# Mini-Flank Supra-12th Rib Incision for Open Partial Nephrectomy Compared with Laparoscopic Partial Nephrectomy and Traditional Open Partial Nephrectomy

**DOI:** 10.1371/journal.pone.0089155

**Published:** 2014-02-21

**Authors:** Hang Wang, Lin Zhou, Jianming Guo, Li'an Sun, Qilai Long, Yong Ma, Li Zhang, Zongming Lin, Tongyu Zhu, Guomin Wang

**Affiliations:** Department of Urology, Zhongshan Hospital, Fudan University, Shanghai, China; University of British Columbia, Canada

## Abstract

**Purpose:**

The purpose of this study was to report our approach of partial nephrectomy (PN) using a supra-12th rib mini-flank incision. We compared mini-incision open partial nephrectomy (MI-OPN) with open partial nephrectomy (OPN) and laparoscopic partial nephrectomy (LPN) to verify whether MI-OPN can be an alternative to OPN and LPN.

**Methods:**

This was a retrospective single-center study including 194 patients who underwent partial nephrectomy (PN) between February 2005 and December 2010. Demographic, perioperative, and complication data were compared among the MI-OPN group, OPN group and LPN group.

**Results:**

No statistical differences were reported in either group for age, sex, BMI, tumour side (right or left kidney), RENAL nephrometry scores, PADUA score and preoperative eGFR. The operative time was longer in LPN group when compared with MI-OPN and OPN group (all P<0.001). The warm ischemia time of LPN group was longer than MI-OPN group (P = 0.032) and OPN group (P = 0.005). The length of stay of LPN group was shorter than OPN group (P = 0.018), but was similar to MI-OPN group (P = 0.094). The incidence of renal artery clamping was lower in OPN group when compared with MI-OPN and LPN group (all P<0.001). More estimated blood loss was found in OPN group when compared with MI-OPN group (p = 0.003) and LPN group (P = 0.014). The overall incidence of postoperative complications was similar.

**Conclusions:**

The approach of MI-OPN can couple the benefits of both minimally invasive and open partial nephrectomy techniques with less estimated blood loss, shorter operative time, shorter length of stay, less postoperative complications, and a smaller incision. MI-OPN may be an effective alternative to laparoscopic or traditional open approaches, which maybe more suitable for the tumors with high RENAL nephrometry score or PADUA score.

## Introduction

Partial nephrectomy (PN) for localised renal cell carcinoma (RCC) has an oncologic outcome similar to that of radical surgery [1]. Patients with low-stage RCC (T1) should undergo nephron-sparing surgery rather than radical nephrectomy whenever possible [2].

Laparoscopic partial nephrectomy (LPN) has been increasingly performed for selected small renal masses, because it has been shown to provide similar oncologic outcomes to that of open partial nephrectomy (OPN) [3], [4]. However, LPN has a higher complication rate compared with open surgery [5]. Open partial nephrectomy currently remains as a standard of care for partial nephrectomy [2].

Historically, three flank approaches have been used for open PN or RN, including subcostal, supracostal, and through the bed of a resected rib.

More recently, Dr. Paul Russo et al. developed a supra-11th rib mini-flank approach for managing renal cortical tumours using either PN or RN. The mini-flank technique couples optimum anatomical exposure and a better aesthetic outcome with a minimum risk of long-term wound complications [6].

Similarly, we developed a supra-12th rib mini-flank approach for managing renal lesions in our department. We compared mini-incision open partial nephrectomy (MI-OPN) with open partial nephrectomy (OPN) and laparoscopic partial nephrectomy (LPN) to verify whether MI-OPN can be an alternative to OPN and LPN.

## Materials and Methods

This was a retrospective single-center study including 194 patients who underwent partial nephrectomy (PN) between February 2005 and December 2010. The study was approved by the institutional review board and Independent Ethics Committee of Zhongshan Hospital and all patients gave written informed consent for the surgical procedures. As the study was a review of medical records, the requirement of informed consent for the study was waived. All operations were performed for a solid renal mass with no evidence of locally advanced disease, major vena caval or venous extension, or regional adenopathy. The choice of procedure was based on patient and physician preference. All PNs were performed by a team of four experienced surgeons. All OPNs and LPNs were performed by four experienced surgeons including Dr. Wang. All MI-OPNs were performed by Dr. Wang.

Patient demographics (sex, age), clinical characteristics (body mass index, hospital stay, estimated glomerular filtration rate [eGFR]), surgical characteristics (operative time, estimated blood loss, Warm ischemia time), postoperative complications, and pathologic characteristics were compared for each technique. The RENAL nephrometry score [7] and PADUA score [8] were used to assess the characteristics of the tumours. Complication data were prospectively collected using the Clavien scale [9]. The eGFR was calculated with MDRD equation [10].

### 2.1 Surgical technique

#### Surgical Technique: MI-OPN

A formal flank position was used. The incision extended from anterior axillary line to the posterior axillary line above the 12th rib, which was off the tip of the 12th rib. The length of incision was about 6∼8 cm. The skin and subcutaneous tissues were opened to expose the external abdominal oblique muscle and latissimus dorsi muscle.

The latissimus dorsi, external oblique and internal oblique muscles were transected. Care must be taken opening the internal oblique in order to avoid damaging the subcostal nerve, which lies between the internal abdominal oblique muscle and the underlying transversalis abdominus. The lumbodorsal fascia is opened at the tip of the rib to avoid both peritoneum and pleura. The transversalis fascia was divided carefully to violating the pleural cavity by displacing the pleura away with the fingers.

The intercostal muscles above the12th rib were retracted to exposure of the suprarenal areas. Gerota's fascia was then bluntly developed exposing the kidney, ureter, and ipsilateral great vessel (vena cava, aorta). Once mobilized from the surrounding fat, the kidney was carefully inspected visually or with US to determine the depth and proximity of the tumour to the major renal vessels and collecting system, and to search for unsuspected satellite tumours that might have escaped preoperative clinical detection. After the renal arteries were clamped with endobulldog clamps, the tumor was removed with a small surrounding margin of grossly normal renal parenchyma. Deep renal tumour specimen margins must be confirmed to be negative for tumour by frozen-section analysis.

After excision of the tumor, remaining transected blood vessels on the renal surface were secured with 3-0 Vicryl sutures. Residual collecting system defects were similarly closed with continuous 4-0 Vicryl sutures. With haemostasis achieved, the renal artery was unclamped. The remaining renal parenchyma was closed with 2-0 Dexon sutures in figure-of-U. At the same time, soluble hemostatic gauzes were placed under the sutures in case of cutting the renal parenchyma.

#### Surgical Technique: OPN

The technique of traditional OPN was similar with MI-OPN with retroperitoneal procedure. Routinely our team performed open partial nephrectomy with cold ischemia and without resecting 12th ribs. Differently, access was provided with the patient in a full-flank position via an extended lumbar incision above or under the 12th rib. As for small lesions, two experienced surgeons preferred to perform OPN without renal hilar clamping and sometimes with pinching the renal or nothing at all.

#### Surgical Technique: LPN

Standard laparoscopic PN procedures were performed. In all patients a transperitoneal approach was used. The kidney was mobilized within Gerota's fascia and defatted, maintaining perirenal fat over the tumour. The renal arteries were clamped with endobulldog clamps. The tumour was excised with cold scissors.

The specimen was immediately put in an Endocatch bag, which was removed at the end of the procedure. Transected blood vessels on the renal surface were secured with 3-0 Vicryl sutures. The renal parenchymal repair was performed with interrupted sutures with 2.0 Vicryl stitches or running sutures. The running suture was locked at both tail ends using Hem-o-lock clips. The collecting system was repaired with Interrupted 3.0 Vicryl stitches.

### 2.2 Statistical analysis

The Wilcoxon and Kruskal-Wallis tests were used to compare nonparametric continuous data, and the Chi-Square tests and Fisher's exact test tests were used to compare nominal data. Multivariable analyses were applied to estimated blood loss and operative time. Predictors of interest for estimated blood loss and operative time included age, RENAL score, individual surgeon (four surgeons), and surgical approach (MI-OPN, LPN and OPN). All analyses were performed using IBM SPSS Statistics v.19 (IBM Corp., Armonk, NY, USA), with p<0.05 considered statistically significant.

## Results

**Figure 1:**
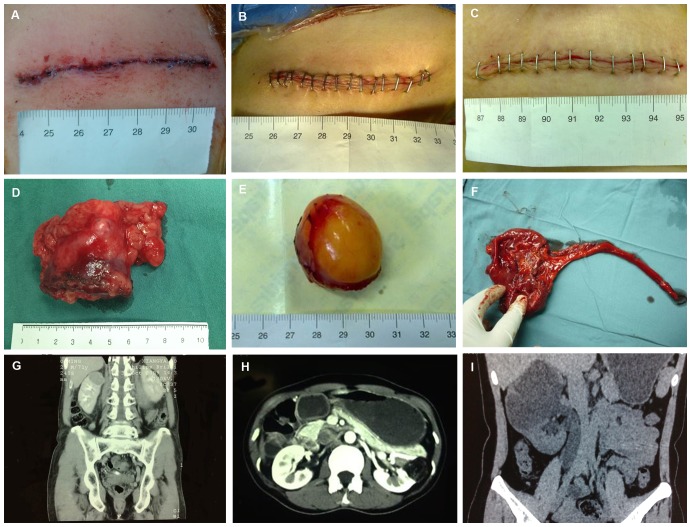
The supra-12th rib mini-flank approach for managing renal lesions. Renal clear cell carcinoma in the upper pole of the right side (A, D, G): The length of incision after MI-OPN (A); The length of the lesion after MI-OPN (D); Coronal reconstruction demonstrates a renal mass in the upper pole of the right kidney (G). Renal angiomyolipoma in the left side (B, E, H): The length of incision after MI-OPN (B); The length of the lesion after MI-OPN (E); Axial computed tomography (CT) scan shows a mass in the left kidney with low-density dark areas (H). Duplex kidney in the right side (C, F, I): C The length of incision after MI-OPN (C); The length of the lesion after MI-OPN (F); Coronal reconstruction demonstrates the right duplex kidney (I).

All the MI-OPN and OPN procedures were completed successfully. Three patients in LPN group were converted to open procedure, mainly because of uncontrollable bleeding. The incision length of MI-OPN was 8.06±0.88 (SD) cm (Fig. 1a–c).

The baseline characteristics of the patients are shown in Table 1. No statistical differences were reported in either group for age, sex, BMI, tumour side (right or left kidney), RENAL nephrometry scores, PADUA score and preoperative eGFR. The mean RENAL nephrometry score for MI-OPN, OPN and LPN were 6.13 (2.20), 6.42 (1.93) and 5.60 (2.02), respectively. The mean PADUA score for MI-OPN, OPN and LPN were 7.75 (2.00), 8.03 (1.83) and 6.87 (1.78), respectively.

**Table 1:** Demographic and clinical characteristics of patients.

Variable	MI-OPN	OPN	LPN	p (MI-OPN vs OPN)	p (MI-OPN vs LPN)	p (LPN vs OPN)
**N**	41	111	42			
**Mean age ± SD (range)**	48.9±13.1 (23–84)	52.8±11.8 (24–84)	50.3±14.4 (25–87)	0.052	0.622	0.228
**Sex, n (%)**				0.204	0.857	0.682
** Male**	29 (70.7%)	67(60.4%)	28(66.7%)			
** Female**	12 (29.3%)	44(39.6%)	14(33.3%)			
**Mean BMI (kg/m^2^), mean ±SD (range)**	23.62±4.11 (22.7–27.8)	23.9±5.23 (22.9–34.6)	23.42±3.34 (23.3–32.7)	0.06	0.476	0.721
**Lesion side, n (%)**				0.818	0.624	0.303
** Left**	22 (53.7%)	62 (55.9%)	19 (45.2%)			
** Right**	19(46.3%)	49(44.1%)	23(54.8%)			
**RENAL nephrometry score, mean ± SD**	6.13±2.20 (4–11)	6.42±1.93 (4–11)	5.60±2.02 (4–10)	0.129	0.503	0.480
**PADUA score, mean±SD**	7.75±2.00 (6–13)	8.03±1.83 (6–14)	6.87±1.78 (6–12)	0.126	0.217	0.888
**Preoperative eGFR (mL/min/1.73 m^2^), mean± SD, (range)**	90.8±23.0 (45.5–150.0)	93.2±22.5 (33.4–153)	92.3±22.2 (56–154.0)	0.291	0.625	0.758

VS  =  versus; MI-OPN =  mini-incision open partial nephrectomy; OPN =  open partial nephrectomy; LPN =  laparoscopic partial nephrectomy; BMI =  body mass index; eGFR  =  estimated glomerular filtration rate; SD =  standard deviation.

Surgical features are shown in Table 2. The operative time was longer in LPN group (131.5 min) when compared with MI-OPN (98 min, p<0.001) and OPN group (99.2, p<0.001). There were significantly lower rates of renal artery clamping in OPN group compared with MI-OPN group (OPN 16.2%; MI-OPN 85.4%; p<0.001) and LPN group (OPN 16.2%; LPN 95.2%; p<0.001). The warm ischemia time was longer in LPN group (27.7 min) compared with MI-OPN group (23.9 min, p = 0.032) and OPN group (21.7 min, p = 0.005). More blood loss was found in OPN group (226.3 ml) compared with MI-OPN group (103.4 ml, p = 0.003) and LPN group (119.3 ml, p = 0.014). Fewer patients in LPN group (14.3%) underwent the suture of the collecting system compared with MI-OPN group (36.6%, p = 0.019) and OPN group (29.7%, p = 0.043). The amount of drainage was less in LPN group (81.8 ml) when compared with MI-OPN (167.6 ml, P<0.001) and OPN group (149.3 ml, p<0.001). The length hospital stay of LPN group (6.65 d) was shorter than OPN group (8.2 d, p = 0.018), but was similar with MI-OPN group (6.8 d, p = 0.094). At the 1–3 years follow-up, there was no significant difference in eGFR between the groups. No kidney was postoperatively lost because of warm ischaemic injury. The pathological diagnosis was similar between the groups (Table 3). The majority of the tumors were T1a with not significant differences.

**Table 2:** Surgical features stratified by group.

Variable	MI-OPN	OPN	LPN	p (MI-OPN vs OPN)	p (MI-OPN vs LPN)	p (LPN vs OPN)
**OT (min) ±SD (range)**	98±21.2 (70–140)	99.2±33.8 (60–260)	131.5±47.9 (60–300)	0.841	<0.001	<0.001
**Renal artery clamping, n(%)**	35(85.4%)	18(16.2%)	40(95.2%)	<0.001	0.129	<0.001
**WIT (min) ±SD (range)**	23.9±5.1 (12–37)	21.7±7.4 (7–40)	27.7±9.5 (15–50)	0.13	0.032	0.005
**EBL (ml) ±SD (range)**	103.4±88.3 (20–500)	226.3±264.8(10–3000)	119.3±150.3(20–800)	0.003	0.569	0.014
**Suture of the collecting system**	15 (36.6%)	33 (29.7%)	6 (14.3%)	0.358	0.019	0.043
**Amount of drainage (ml) ±SD (range)**	167.6±113.9(30–520)	149.3±127.8(0–848)	81.8±59.1 (20–230)	0.395	<0.001	<0.001
**LOS (d) ±SD (range)**	6.8±2.1 (5–17)	8.2±3.9 (3–39)	6.65±2.5 (3–16)	0.094	0.374	0.018
**GFR at the 1–3 years follow-up (mL/min/1.73 m^2^), mean ± SD, (range)**	81.8±29.0 (40.0–129.0)	84.4±28.7 (28.2–138.0)	81.5±24.7 (62.0–107.0)	0.236	0.348	0.846

OT = operative time; WIT = Warm ischemia time; LOS =  length of stay; EBL =  estimated blood loss.

**Table 3:** Postoperative histopathology results.

Variable	MI-OPN	OPN	LPN	p (MI-OPN vs OPN)	p (MI-OPN vs LPN)	p (LPN vs OPN)
**Pathological diagnosis**				0.296	0.927	0.351
**RCC, n (%)**	24(58.5%)	74(67%)	25(59.5%)			
**Benign, n (%)**	17(41.5%)	37(33%)	17(40.5%)			
**Renal cell carcinoma**						
**Clear cell, n (%)**	19(46.3%)	61(55%)	21(50.0%)	0.296	0.739	0.535
**Papillary, n (%)**	2(4.8%)	5(4.5%)	2(4.8%)	1.000	1.000	1.000
**Chromophobe, n (%)**	3(7.3%)	5(4.5%)	2(4.8%)	0.435	0.676	1.000
**Other, n (%)**	0(0.0%)	3(2.7%)	0(0.0%)	0.594		0.594
**Benign**						
**Angiomyolipoma, n (%)**	9(22%)	28(25.2%)	10(23.8%)	0.603	0.854	0.787
**Complicatedcyst, n (%)**	6(14.6%)	4(3.6%)	7(16.7%)	0.002	0.795	0.002
**Duplex kidney, n (%)**	2(4.9%)	5(4.5%)	0(0.0%)	0.684	0.241	0.362
**T stage (RCC), n**				0.451	0.739	0.862
**T1a**	22	65	24	0.538	0.750	0.839
**T1b**	1	6	1	0.704	1.000	0.705
**T2a**	0	1	0	1.000		1.000
**T2b**	0	1	0	1.000		1.000
**T3a**	1	1	0	0.289	0.494	1.000

RCC = renal cell carcinoma.

The complication rate was 12.2% in the MI-OPN, 13.1% in the OPN and 21.4% in LPN group with not significant differences (all p>0.05) (Table 4). There were no grade 4 or 5 complications. After MI-OPN, one patient with urinary leak required an endoscopic intervention (Clavien grade 3), placing a double J ureteral stent. After OPN, four patients (two with delayed renal hemorrhage, and another two with urinary leak) required an endoscopic or surgical intervention (Clavien grade 3). After LPN, two patients (one with delayed renal hemorrhage, and another one with urinary leak) required an endoscopic or surgical intervention (Clavien grade 3).

**Table 4:** Postoperative complications.

Variable	MI-OPN	OPN	LPN	p (MI-OPN vs OPN)	p (MI-OPN vs LPN)	p (LPN vs OPN)
**Complications, n (%)**	5 (12.2%)	15(13.1%)	9(21.4%)	0.872	0.261	0.160
** Clavien I**	3	9	2			
** Clavien II**	1	3	5			
** Clavien III**	1	4	2			

Multivariate analysis of select outcomes (EBL and OT) was performed using the covariates patient age, RENAL score, individual surgeon (four surgeons), and surgical approach (MI-OPN, LPN and OPN) (Table 5). In multivariate analysis the significant predictors of select outcomes (EBL and OT) was RENAL score and surgical approach (all p<0.0005). Individual surgeon and patient age were not associated with estimated blood loss and operative time.

**Table 5:** Multivariate analyses of selected outcomes.

Selected outcomes	p Value
**Estimated blood loss**	
** Age**	0.87
** RENAL score**	<0.0005
** Individual surgeon**	0.089
** Surgical approach**	<0.0005
**Operative time**	
** Age**	0.193
** RENAL score**	<0.0005
** Individual surgeon**	0.055
** Surgical approach**	<0.0005

## Discussion

A flank incision offers extraperitoneal access to the kidney and adjacent structures. The supracostal flank incision is favored at the Lahey Clinic for most modest-sized renal tumors and most partial nephrectomies [11]. More recently, a modified mini-flank supra-11th rib incision has been described as a safe, effective approach to radical or partial nephrectomy for renal cortical tumors [6]. Differently, we use a lower incision at the level of supra-12th rib. For partial nephrectomy the level of incision is determined by the position of the kidney in relation to the ribs as seen on preoperative radiographic studies and by the location and size of the tumor. Typically, for a lower pole tumor a supra-12th rib incision is adequate. For mid and upper pole tumors, a supra-11th incision is often satisfactory. But in our experience, a supra-12th rib incision is adequate for both lower and upper pole tumors.

Open nephrectomy through a flank incision has been the preferred approach to renal cancer for many surgeons. This method provides excellent exposure with minimal disturbance of abdominal viscera. The traditional open partial nephrectomy utilized a large flank incision with or without resection of the eleventh rib. Although this approach provided wide exposure to the kidney and retroperitoneum, patients complained of significant postoperative pain, a prolonged recovery, and for many an uncomfortable and unsightly flank bulge usually from muscle atony from nerve damage as opposed to fascial hernia. Flank approaches can result in injury to the intercostal nerves with denervation and paresis of the flank musculature, leading to chronic postoperative pain or flank bulge in 3% to 49% of patients [12], [13].

Canadian investigators described 70 patients who underwent formal flank or thoracoabdominal incision for RN (69%) or PN (31%) [13]. 49% of patients experienced a flank bulge. This study indicated that the rate of flank bulge formation after flank incision for nephrectomy for renal tumors had been substantially underreported in the previous literature. More recently, Dr. Paul Russo et al. reported that 1.8% [14] to 4% [6] patients who underwent supra-11th rib mini-flank incision had complications related to the operative site (i.e. herniation, flank bulge). In our study, MI-OPN can safely provide optimum anatomical exposure without rib exposure with decreased estimated blood loss (MI-OPN 103 ml; OPN 226; p = 0.003) and length of stay (MI-OPN 6.8 d; OPN 8.2 d; p = 0.094) as well as a better cosmetic result compared with OPN. In MI-OPN group, no patient complained of a flank bulge persisting more than 1 year after surgery. Recently, Dr. Paul Russo et al. encouraged early ambulation (walking a mile around the hospital ward on postoperative day 1), which made a 2-day hospital stay achievable in most patients [15]. Two-day hospital stay is a great advantage and worth trying.

The amount of drainage was more in MI-OPN group than LPN group (167.6 ml vs. 81.8 ml, p<0.001). The transperitoneal approach of LPN may be the reason of less amount of drainage, which could be absorbed by the peritoneum. The warm ischemia time (WIT) and operative time (OT) was lower in MI-OPN group than LPN group (WIT: 23.9 min vs. 27.7 min, p = 0.032; OT: 98 min vs. 131.5 min, p<0.001). In multivariate analysis the significant predictors of select outcomes (EBL and OT) was RENAL score and surgical approach (all p<0.0005). The lower WIT and OT in MI-OPN group can be explained by precise and rapid hemostasis. Although no significant difference was found, there was lower rate of postoperative complications in MI-OPN group (12.2%) than LPN group (21.4%). In the most experienced center of Hopkins and Cleveland Clinic, the complication rate of LPN is up to 18% and 28% [5], [6]. The incidence of renal artery pseudoaneurysm is higher after minimally invasive partial nephrectomy than after an open approach [17]. In addition, laparoscopic approaches require an incision to retrieve the specimen, which is susceptible to similar complications as those in traditional OPN. The difficulty in learning this method and the demand for extensive training at specialized centers are limitations to LPN [18], [19]. Nadu et al [20] and Frank et al [21] both compared central vs. peripheral tumours treated with laparoscopic partial nephrectomy and reported increased warm ischaemia time and length of hospital stay associated with central tumours. Compared with LPN, maybe MI-OPN is more suitable for the tumors with high RENAL nephrometry score or PADUA score.

The approach of MI-OPN can couple the benefits of both minimally invasive and open partial nephrectomy techniques. MI-OPN represents an alternative approach that can be easily adopted by practicing urologists. The other advantages include less estimated blood loss compared with OPN, and shorter warm ischemia time and shorter operative time compared with LPN. Although no statistical difference was found, the rate of postoperative complications of MI-OPN was similar with OPN and lower than LPN.

We recognize the shortcomings of the current study as a retrospective, nonrandomized comparison, which led to an inherent selection bias that could not be overcome. Moreover, the OPN group was at higher risk for operative intervention because of a preponderance of larger tumors. Furthermore, limited sample size might have reduced statistical power in subgroup analysis.

## Conclusion

The approach of MI-OPN can couple the benefits of both minimally invasive and open partial nephrectomy techniques with less estimated blood loss, shorter operative time, shorter length of stay, less postoperative complications, and a smaller incision. MI-OPN may be an effective alternative to laparoscopic or traditional open approaches, which maybe more suitable for the tumors with high RENAL nephrometry score or PADUA score.
